# Fixed point-based stability analysis of climate and Langevin models

**DOI:** 10.1371/journal.pone.0327488

**Published:** 2025-07-03

**Authors:** Syed Khayyam Shah, Waleed Eltayeb Ahmed, Ishraq Alabdi, Ayman Alahmade, Khaled Aldwoah, Eltigani I. Hassan

**Affiliations:** 1 Department of Sustainable Environment and Energy Systems (SEES), Middle East Technical University, Northern Cyprus Campus, Kalkanli, Guzelyurt, Mersin 10, Turkey; 2 Department of Mathematics and Statistics, Imam Mohammad Ibn Saud Islamic University (IMSIU), Riyadh, Saudi Arabia; 3 Department of Mathematics, Hajjah University, Hajjah, Yemen; 4 Department of Mathematics, College of Science, Taibah University, Medina, Saudi Arabia; 5 Department of Mathematics, Faculty of Science, Islamic University of Madinah, Madinah, Saudi Arabia; Tezpur University, INDIA

## Abstract

In this manuscript, the existence and uniqueness of solutions to equations associated with climate change are discussed. For this purpose, we utilize some results from the existing literature to investigate the behavior of these equations. Additionally, the role of fixed point theory in emphasizing the importance of proving the stability and consistency of the models is explored. Several definitions and results, such as the *F*-contraction, α-*F*-contraction, rational type (ψ,ϕ)-contraction, and Geraghty type contraction, are recalled from the existing literature to illustrate their theoretical foundations and practical applications.

## 1 Introduction

Mathematics serves as a highly efficient and potent tool for understanding the world and addressing intricate challenges across diverse scientific fields, engineering, and technology [1–3]. In this context, the crucial role of mathematics cannot be overstated in addressing planetary issues, particularly the connection between society and nature [4,5]. Humans engage in various activities through interactions with their environment. Humans influence the environment by altering its properties, while environmental conditions, in turn, impact human health and survival capabilities. The present climate change, in contrast to other instances, is anthropogenic and marked by an unparalleled acceleration in the global mean surface temperature (GMST) [6,7]. In [8], it is indicated that from 1880, the GMST has risen upto 0.07 C by an average per decade. In the 21st century, notably in the first two decades, the growth rate of the GMST was 0.17 C for a decade, which is more than twice the average rate. A notable indicator of human activities is the considerable rise in atmospheric carbon dioxide (CO_2_) concentrations. This greenhouse gas, a by-product of the combustion of fuels, turns out to be the primary culprit for global warming. This greenhouse gas, a by-product of fuel combustion, is considered the primary driver of global warming.

Global warming has impacted climate across nearly all regions of our planet; however, the extent of this effect varies considerably by region [6,7]. Global and regional climate changes evidently influence individuals and their activities [9,10]. Evaluating climate hazards is a necessary step in determining how humanity may be affected by changing climate conditions [11]. Mathematics and its methodologies are essential in climate study [12,13]. It can be asserted assuredly that mathematics is the sole discipline that enables us to measure and forecast the impacts of external natural and man-made disturbances on the dynamics of the climate system. Quantitative research fundamentally relies on mathematical representations of the systems and objects being studied. In this context, we see that replicating climate and forecasting its alterations due to external influences, unlike traditional physics problems, possesses a fundamental characteristic stemming from the inability to conduct comprehensive direct physical experiments. This highlights the importance of stability of the solutions of the equations associated with climate change models is of great importance.

The equation,

$$\frac{d\nu }{dt}=\Gamma (\nu ,\alpha ,F)\;\nu \in R^{n},\;\nu (0)=\nu_{0},\;t\geq 0,$$ (1)

denotes the mathematical representation of the Earth’s Climate System (ECS) [14–16], encompassing its dynamic development. Here, ν is a vector of state variables that delineate the system’s state at time *t*, while $\nu_{0}$ represents its initial state. The parameter vector $\alpha \in {\mathbb{R}}^{m}$ governs the complex traits of the system, and F symbolizes external forcing, such as solar radiation or anthropogenic impacts. This system of three-dimensional nonlinear partial differential equations captures the intended connection between internal climate parameters and external influences, offering a deterministic, semi-dynamic formulation.

The Langevin equation draws the interest of researchers, as it possesses significant importance in stochastic dynamics. This equation is commonly utilized to demonstrate the systems affected by deterministic forces and random fluctuations [14]:

$$\frac{dx}{dt}+\beta x=g(t),$$ (2)

where *x* represents the state variable, β stands for the damping effect with *g*(*t*) means the external influence on the system.

Fixed point theory (FPT) is significant for analyzing mathematical models that represent climate change. FPT provides the groundwork for showcasing the stability and consistency of solutions for differential and integral equations associated with these models. In the models of Climate proper simulation, good mathematical modeling is essential for stability and predictability, notably with the help of fixed-point results like Banach’s Fixed Point Theorem, etc. This indicates that, at a fixed point, we may determine if a stable solution exists to which this system will converge under particular conditions. These methods ensure the existence and stability of solutions, which is essential for analysis and informed decision-making.

In this direction, this manuscript examines the worth of the FPT in investigating the stability of those equations that are in association with the climate change system. In this direction, some important contractions such as *F*-contraction, α-*F*-contraction, rational type (ψ,ϕ)-contraction, and Geraghty type contraction have been recalled and utilized to define the existence and uniqueness criteria for those equations. The objective is to emphasize the fundamental characteristics that guarantee the practical effectiveness of these models in the disciplines of meteorology and climatology by conducting a thorough analysis and applying fixed-point theorems from the existing literature.

## 2 Preliminaries

Onward, we will denote the fixed point as (FP) and the unique solution as (Uni-Sol).

Some important definitions in this sequel are listed below:

**Definition 2.1.** [17] *For a non-empty set* $M_{b}$, *define* $b_{m}:M_{b}\times M_{b}\to [0,+\infty )$ *with* 𝔟≥1, *if the below hold:*

1. $b_{m}(q,s)=0\Rightarrow q=s,$ $\forall q,s\in M_{b}$.
2. $b_{m}(q,s)=b_{m}(s,q),$ $\forall q,s\in M_{b}$.
3. $b_{m}(q,s)\leq 𝔟[b_{m}(q,t)+b_{m}(t,s)]$, $\forall q,s,t\in M_{b}$.

*Then, we labeled* $\left(M_{b},b_{m}\right)$ *as* 𝔟*-metric space.*

**Definition 2.2.** [18] *Suppose* $ϝ:{\mathbb{R}}^{+}\to \mathbb{R}$ *represent a mapping which fulfill the below conditions:*

(1) ϝ *is strictly increasing, i.e., when* σ>∋, *for all* $\sigma ,∋\in {\mathbb{R}}^{+}$ *implies to* *ϝ* ( *σ* ) < *ϝ* ( *∋* ) .
(2) *For each sequence* $\{\sigma_{\eta }{\}}_{\eta \in \mathbb{N}}$ *of positive terms,* $\lim_{\eta \to +\infty }\sigma_{\eta }=0$ iff $\lim_{n\to +\infty }ϝ(\sigma_{\eta })=-\infty$.
(3) *There is* t∈(0,1) *in a sense that* $\lim_{\sigma \to 0^{+}}\sigma^{t}ϝ(\sigma )=0$.

**Example 2.1.** *[19] For* $ϝ:{\mathbb{R}}^{+}\to \mathbb{R}$:

1. ϝ(B)=lnB,B>0,
2. ϝ(B)=lnB+B,B>0,
3. $ϝ(B)=\frac{-1}{\sqrt{B}},\;B>0$,
4. $ϝ(B)=\ln(B^{2}+B),\;B>0$,

*are all the examples of* ϝ.

**Definition 2.3.** *[3] Let* $\left(M_{b},b_{m}\right)$ *represent a* 𝔟*-metric space, and let* $\left\{v_{n}\right\}$ *be a sequence in* $M_{b}$ *with* $e\in M_{b}$. *Then the sequence* $\left\{v_{n}\right\}$ *is referred to be:*

*(a) convergent in* $\left(M_{b},b_{m}\right)$ *and converges to e when, for every* ε>0, *there exists* $n_{0}\in N$ *in a way that* $b_{m}(v_{n},e)<\varepsilon$ *for all n*>*n*_0_, *and will be expressed as* $\lim_{n\to \infty }v_{n}=e$ *or* $v_{n}\to e$ *as* n→∞.

*(b) Cauchy if, for every* ε>0, *there is some* N=𝒩(ε)∈N *such that* $b_{m}(v_{m},v_{n})<\varepsilon$ *for all* m,n≥N.

**Definition 2.4.** *[3] Let* $\left(M_{b},b_{m}\right)$ *denote a* 𝔟*-metric space then, it is labeled as complete if every Cauchy Sequence in* $M_{b}$ *converges.*

**Definition 2.5.** *[19] For a non-empty set* 𝔐, *a mapping* W:𝔐→𝔐 *will be* α*-admissible when there exists a function* $\alpha :𝔐\times 𝔐\to {\mathbb{R}}^{+}$ *in a sense*


q,y∈𝔐,α(q,y)≥1⟹α(Wq,Wy)≥1.

**Definition 2.6.** *[20] Suppose* $\left(𝔐,d_{m}\right)$ *express the structure metric space, a mapping* B:𝔐→𝔐 *will be termed* Ćirić *type generalized* ϝ*-contraction when there is* τ>0 *in a sense that,*

$$\left[d_{m}(B\nu ,B\omega )>0\Rightarrow \tau +ϝ(d_{m}(B\nu ,B\omega ))\leq ϝ(M(\nu ,\omega ))\right],\;\forall \nu ,\omega \in 𝔐,$$ (3)

*while*

$$M(\nu ,\omega )=\max\left\{d_{m}(\nu ,\omega ),d_{m}(\nu ,B\nu ),d_{m}(\omega ,B\omega ),\frac{1}{2}[d_{m}(\nu ,B\omega )+d_{m}(\omega ,B\nu )]\right\}.$$

**Theorem 2.1.** *[20] Suppose* $\left(𝔐,d_{m}\right)$ *represent a complete metric space and* B:𝔐→𝔐 *is a Ćirić type generalized* ϝ−contraction. *When B or* ϝ *is continuous, then B admits a unique FP.*

**Definition 2.7.** *[19] Suppose* $\left(𝔐,d_{m}\right)$ *express a metric space, a mapping* B:𝔐→𝔐 *be labeled as modified* α−ϝ*-contraction. When for functions* ϝ *and* α:𝔐×𝔐→(−∞,0)∪(0,+∞) *some* τ>0 *exists such a way that* $d_{m}(B\nu ,B℘)>0$ *and,*

$$\tau +\alpha (\nu ,℘)ϝ(d_{m}(B\nu ,B℘))\leq ϝ(M_{a}(\nu ,℘)),$$

*where*

$$M_{a}(\nu ,℘)=\max\{d_{m}(\nu ,℘),d_{m}(\nu ,B\nu ),d_{m}(℘,B℘),\frac{d_{m}(\nu ,B\nu )d_{m}(\nu ,B\nu )+d_{m}(℘,B\nu )d_{m}(℘,B℘)}{d_{m}(℘,B\nu )+d_{m}(\nu ,B℘)},\;\frac{d_{m}(\nu ,B\nu )d_{m}(℘,B℘)}{d_{m}(\nu ,℘)+d_{m}(\nu ,B℘)+d_{m}(℘,B\nu )}\}.$$

**Theorem 2.2.** *[19] Suppose* $\left(𝔐,d_{m}\right)$ *express a complete metric space, when a continuous mapping* B:𝔐→𝔐 *is* α−ϝ−weak *contraction and* α−admissible, *with* $\alpha ({℘}_{0},B{℘}_{0})\geq 1$. *Then B possesses a unique FP.*

**Definition 2.8.** *[21] Suppose a family of functions* 𝒲, *as* $\alpha_{c}:{\mathbb{R}}^{+}\to [0,1)$ *with the property of:*


$$\lim_{n\to \infty }\alpha_{c}(z_{n})=1⟹\lim_{n\to \infty }z_{n}=0.$$

**Theorem 2.3.** [21] *Suppose* $\left(𝔐,d_{m}\right)$ *represent a complete metric space. Suppose* C:𝔐→𝔐 *be a self-map fulfilling the below:*

$$d_{m}(Ce,Ck)\leq \alpha_{c}(d_{m}(e,k))d_{m}(e,k),\;\forall e,k\in 𝔐.$$

*where* $\alpha_{c}\in 𝒲$. *So this will ensure a unique FP for C*.

**Definition 2.9.** *[22] For b-metric space* $\left({𝔐}_{b},b_{m}\right)$ *with* 𝔟≥1, *suppose* ${ℬ}_{𝔟}$ *is a set representing all the functions that are defined as* $\beta :[0,\infty )\to \left[0,\frac{1}{𝔟}\right)$ *that satisfy the below*

$${lim sup}_{n\to \infty }\beta (z_{n})=\frac{1}{𝔟}⟹z_{n}\to 0\;\text{as}\;n\to \infty .$$

**Theorem 2.4.** *[22] Suppose* $\left({𝔐}_{b},b_{m}\right)$ *represents a complete b-metric space along* 𝔟≥1. *Moreover, let* $𝒫:M_{b}\to M_{b}$ *denotes a self-map. If there exists* $\beta \in {ℬ}_{𝔟}$ *in a sense that*

$$b_{m}(𝒫𝔲,𝒫y)\leq \beta \left({ℳ}_{c}(𝔲,y)\right){ℳ}_{c}(𝔲,y),$$

*where*

$${ℳ}_{c}(𝔲,y)=\max\left\{b_{m}(𝔲,y),b_{m}(𝔲,𝒫𝔲),b_{m}(y,𝒫y),\frac{1}{2𝔟}\left(b_{m}(𝔲,𝒫y)+b_{m}(y,𝒫𝔲)\right)\right\},$$

*then* 𝒫 *ensures a unique FP.*

Consider the following two functions,

ψ:[0,∞)→[0,∞) a monotone non-decreasing and continuous function with ψ(𝔤)=0 iff 𝔤=0.

φ:[0,∞)→[0,∞) is a lower semi-continuous function in a way that φ(𝔤)=0 iff 𝔤=0.

**Theorem 2.5.** *[23] Suppose* $\left(𝔐,d_{m}\right)$ *represent a complete metric space and suppose* B:𝔐→𝔐 *represent a mapping. Let*

$$\psi (d_{m}(B𝔲,By))\leq \psi (M_{b}(B𝔲,By))-\varphi (M_{b}(B𝔲,By)),\;\forall \;𝔲,y\in 𝔐,$$ (4)

*whereas*

$$M_{b}(𝔲,y)=\max\{d_{m}(𝔲,y),\;d_{m}(𝔲,B𝔲),\;d_{m}(y,By),\frac{d_{m}(y,B𝔲)+d_{m}(𝔲,By)}{2},\;\;\frac{d_{m}(𝔲,B𝔲)+d_{m}(y,By)}{2},\;d_{m}(y,By)\left(\frac{1+d_{m}(𝔲,B𝔲)}{1+d_{m}(𝔲,y)}\right),\;d_{m}(𝔲,B𝔲)\left(\frac{1+d_{m}(y,B𝔲)}{1+d_{m}(𝔲,y)}\right)\}.$$ (5)

*Then there will be a unique point* ${𝔲}^{*}\in 𝔐$ *in a sense that* ${𝔲}^{*}=B{𝔲}^{*}$.

## 3 Existence results for ECS’s equation

In this section, various important contractions from the fixed point theory will be utilized to investigate the stability of the equation associated with ECS.

$$\frac{d\nu }{dt}=\Gamma (\nu ,\alpha ,F),\;\nu \in R^{n},\;\;\nu (0)=\nu_{0},\;\;t\geq 0,$$ (6)

where ν represents the vector of state variables that define the system’s state at certain points in time *t*, $\nu_{0}$ represents a specified initial condition of a system, *n* denotes the dimension for a dynamical system, $\alpha \in R^{m}$ express the *m*-dimensional parameter vector along F representing external forcing. By transforming the differential equation into the integral form, the model equation will become:

$$\nu (t)=\nu_{0}+\int_{0}^{t}\Gamma (\nu ,\alpha ,F)\;d℘,\;t\geq 0.$$ (7)

In this direction, we aim to establish an existence result for investigating the solution of the integral equation of the type (7), utilizing Definition 2.6 and Theorem 2.1 from the existing literature.

Now, our objective is to demonstrate the existence of a solution to the integral Eq (7) in the given space and also to verify the uniqueness of the solution.

Let $𝔐=C[t_{0},\gamma ]$ denote the set of all real-valued continuous functions defined on $\left[t_{0},\gamma \right]$. We define $d_{m}:𝔐\times 𝔐\to {\mathbb{R}}_{+}$ as follows:

$$d_{m}(𝔲,y)=|𝔲(t)-y(t)|,\;\text{for all }𝔲,y\in 𝔐.$$ (8)

Evidently, $\left(𝔐,d_{m}\right)$ represent a complete metric space.

Here is our first new result.

**Theorem 3.1.** *Suppose the following presumptions are fulfilled:*

***(b)** The function* $\Gamma :[t_{0},\gamma ]\times [t_{0},\gamma ]\times \mathbb{R}\to \mathbb{R}$ *is continuous on* $\left[t_{0},\gamma \right]$ with $[t_{0},\gamma {]}^{2}$, *and* $[t_{0},\gamma {]}^{2}\times \mathbb{R}$, *correspondingly.*
***(c)*** ∀ $\alpha ,\nu ,𝔣\in [t_{0},\gamma ]$ and for every 𝔲,y∈𝔐,
  $$|\Gamma (\nu_{1},\alpha_{1},{𝔣}_{1})\;-\Gamma (\nu_{2},\alpha_{2},{𝔣}_{2})\;|\leq \frac{M(𝔲,y)}{e^{\tau }(1+t)},$$
*where* M(𝔲,y) *is defined in Definition 2.6.*

*Then, (7) will have a Uni-Sol.*

*Proof*: *Define a map* Θ:𝔐→𝔐

$$\Theta \nu (t)=\nu_{0}+\int_{0}^{t}\Gamma (\nu ,\alpha ,𝔣)\;d℘\;t\geq 0.$$ (9)

*The existence of a FP of* Θ *in (9) will be evidently the Uni-Sol of the Eq (7).*

*Now utilizing (8) and the above presumptions **(b)** to **(c)***.

$$|\Theta \nu_{1}(t)-\Theta \nu_{2}(t)|=\left|\nu_{0}+\int_{0}^{t}\Gamma (\nu_{1},\alpha_{1},{𝔣}_{1})\;d℘-\nu_{0}-\int_{0}^{t}\Gamma (\nu_{2},\alpha_{2},{𝔣}_{2})\;d℘\right|\;=\left|\int_{0}^{t}\left(\Gamma (\nu_{1},\alpha_{1},{𝔣}_{1})-\Gamma (\nu_{2},\alpha_{2},{𝔣}_{2})\right)\;d℘\right|\;\leq \int_{0}^{t}\left|\Gamma (\nu_{1},\alpha_{1},{𝔣}_{1})-\Gamma (\nu_{2},\alpha_{2},{𝔣}_{2})\right|d℘\;\leq \frac{M(𝔲,y)}{e^{\tau }(1+t)}\int_{0}^{t}d℘\;=\frac{M(𝔲,y)}{e^{\tau }(1+t)}\cdot t\;\leq e^{-\tau }M(𝔲,y).$$

*This means that*

$$d_{m}(\Theta \nu_{1},\Theta \nu_{2})\leq e^{-\tau }M(𝔲,y).$$

*Define* $ϝ:{\mathbb{R}}_{+}\to \mathbb{R}$ *by*

ϝ(𝔚)=ln(𝔚)for𝔚>0.

$$ϝ(d_{m}(\Theta \nu_{1},\Theta \nu_{2}))\leq ϝ(e^{-\tau }M(𝔲,y))\;=\ln(M(𝔲,y))-\tau \ln e.$$

*Implies that,*

$$\tau +ϝ(d_{m}(\Theta \nu_{1},\Theta \nu_{2}))\leq ϝ(M(𝔲,y)).$$

*Thus, all the assumptions of Theorem 2.1 are true. Hence, the Eq (7) will have a Uni-Sol.* ◻

For our next new result let $𝔐=([t_{0},\gamma ],R)$ represent all the real valued continuous functions on $\left[t_{0},\gamma \right]$.

Let us define $d_{m}:𝔐\times 𝔐\to {\mathbb{R}}_{+}$ as follows

$$d_{m}(𝔲,y)=|𝔲(t)-y(t)|,\;\text{for all }𝔲,y\in 𝔐.$$ (10)

Evidently, $\left(𝔐,d_{m}\right)$ represent a complete metric space.

**Theorem 3.2.** *Suppose the following presumptions are fulfilled:*

***(r)** The function* $\Gamma :[t_{0},\gamma ]\times [t_{0},\gamma ]\times \mathbb{R}\to \mathbb{R}$ *is continuous on* $\left[t_{0},\gamma \right]$ *with* $[t_{0},\gamma {]}^{2}$, and $[t_{0},\gamma {]}^{2}\times \mathbb{R}$, *correspondingly.*
***(s)*** ∀ $\alpha ,\nu ,𝔣\in [t_{0},\gamma ]$ *and for every* 𝔲,y∈𝔐,
  $$|\Gamma (\nu_{1},\alpha_{1},{𝔣}_{1})\;-\Gamma (\nu_{2},\alpha_{2},{𝔣}_{2})\;|\leq \frac{M_{a}(𝔲,y)}{e^{\tau }(1+t)},$$
*where* $M_{a}(𝔲,y)$ *is defined in Definition 2.7.*

*Then, (7) will have a Uni-Sol.*

*Proof*: *Define a map* Θ:𝔐→𝔐

$$\Theta \nu (t)=\nu_{0}+\int_{0}^{t}\Gamma (\nu ,\alpha ,𝔣)\;d℘\;t\geq 0.$$ (11)

*The existence of a FP of* Θ *in (11) will be evidently the Uni-Sol of the Eq (*7*).*

*Now utilizing (11) and the above presumptions **(r)** to **(s)***,

$$|\Theta \nu_{1}(t)-\Theta \nu_{2}(t)|=\left|\nu_{0}+\int_{0}^{t}\Gamma (\nu_{1},\alpha_{1},{𝔣}_{1})\;dt-\nu_{0}-\int_{0}^{t}\Gamma (\nu_{2},\alpha_{2},{𝔣}_{2})\;dt\right|\;=\left|\int_{0}^{t}(\Gamma (\nu_{1},\alpha_{1},{𝔣}_{1})-\Gamma (\nu_{2},\alpha_{2},{𝔣}_{2}))\;dt\right|\;\leq \int_{0}^{t}\left|\Gamma (\nu_{1},\alpha_{1},{𝔣}_{1})-\Gamma (\nu_{2},\alpha_{2},{𝔣}_{2})\right|dt\;\leq \frac{M_{a}(𝔲,y)}{e^{\tau }(1+t)}\int_{0}^{t}dt\;=\frac{M_{a}(𝔲,y)}{e^{\tau }(1+t)}\cdot t\;\leq e^{-\tau }M_{a}(𝔲,y).$$

*As a result,*

$$d_{m}(\Theta \nu_{1},\Theta \nu_{2})\leq e^{-\tau }M_{a}(\omega_{1},\omega_{2})$$

*Define* $ϝ:{\mathbb{R}}_{+}\to \mathbb{R}$ *by*

ϝ(𝔚)=ln(𝔚),for𝔚>0.

$$ϝ(d_{m}(\Theta \nu_{1},\Theta \nu_{2}))\leq ϝ(e^{-\tau }M_{a}(𝔲,y))\;=\ln(M_{a}(𝔲,y))-\tau \ln e.$$

*Next define* α:𝔐×𝔐→{−∞}∪[0,+∞) *by*

$$\alpha (\nu_{1},\nu_{2})=\left\{ \begin{array}{c} 1,\;\;\;\text{if}\;d_{m}(\nu_{1},\nu_{2})\geq 0,\\ -\infty ,\;\text{else}. \end{array}\right.$$

*Accordingly,*

$$\tau +ϝ(d_{m}(\Theta \nu_{1},\Theta \nu_{2}))\leq \alpha (\nu_{1},\nu_{2})ϝ(M_{a}(𝔲,y)).$$

*Thus, all the presumptions of Theorem 2.2 hold true. So, the equation (7) will have a Uni-Sol.* ◻

Following the same flow, taking important results of Geraghty-type contractions in the framework of *b*-metric space, we can have novel existence and uniqueness results.

Let ${𝔐}_{b}=[t_{0},\gamma ],R]$ represent all the real valued continuous functions on 𝔐.

Let us define $b_{m}:{𝔐}_{b}\times {𝔐}_{b}\to {\mathbb{R}}_{+}$ as follows

$$b_{m}(𝔲,y)=|𝔲-y{|}^{2},\;\text{for all }𝔲,y\in ({𝔐}_{b},b_{m}).$$ (12)

Evidently, $\left({𝔐}_{b},b_{m},𝔟\right)$ is a complete *b*-metric space with 𝔟≥1.

**Theorem 3.3.** *Suppose the following presumptions are fulfilled:*

***(e)** The function* $\Gamma :[t_{0},\gamma ]\times [t_{0},\gamma ]\times \mathbb{R}\to \mathbb{R}$ *is continuous on* $\left[t_{0},\gamma \right]$ *with* $[t_{0},\gamma {]}^{2}$, *and* $[t_{0},\gamma {]}^{2}\times \mathbb{R}$, *correspondingly.*
***(f)*** ∀ $\nu ,\alpha ,𝔣\in [t_{0},\gamma ]$ *and for every* 𝔲,y∈𝔐,
  $$|\Gamma (\nu_{1},\alpha_{1},{𝔣}_{1})\;-\Gamma_{1}(\nu_{2},\alpha_{2},{𝔣}_{2})\;|\leq \sqrt{\frac{\ln(1+{ℳ}_{c}(\nu_{1},\nu_{2}))}{(1+t)𝔟}},$$
*where* ${ℳ}_{c}(\nu_{1},\nu_{2})$ *is defined in Theorem 2.4.*

*Then, (7) will have a Uni-Sol.*

*Proof*: *Define a map* Θ:𝔐→𝔐

$$\Theta \nu (t)=\nu_{0}+\int_{0}^{t}\Gamma (\nu ,\alpha ,𝔣)\;d℘\;t\geq 0.$$ (13)

*The existence of a FP of* Θ *in (13) will be evidently the Uni-Sol of the Eq (7).*

*Now, utilizing (12), (13) and the above presumptions **(e)** and **(f)***

$$|\Theta \nu_{1}(t)-\Theta \nu_{2}(t){|}^{2}={\left|\nu_{0}+\int_{0}^{t}\Gamma (\nu_{1},\alpha_{1},{𝔣}_{1})\;dt-\nu_{0}-\int_{0}^{t}\Gamma (\nu_{2},\alpha_{2},{𝔣}_{2})\;dt\right|}^{2}\;={\left|\int_{0}^{t}(\Gamma (\nu_{1},\alpha_{1},{𝔣}_{1})-\Gamma (\nu_{2},\alpha_{2},{𝔣}_{2}))\;dt\right|}^{2}\;\leq \int_{0}^{t}{\left|\Gamma (\nu_{1},\alpha_{1},{𝔣}_{1})-\Gamma (\nu_{2},\alpha_{2},{𝔣}_{2})\right|}^{2}dt\;\leq \frac{1}{𝔟(1+t)}\ln(1+{ℳ}_{c}(\nu_{1},\nu_{2}))\int_{0}^{t}dt\;\leq \frac{1}{𝔟}\ln(1+{ℳ}_{c}(\nu_{1},\nu_{2})).$$

*Further, since* 𝔟=2

$$|\Theta \nu_{1}(t)-\Theta \nu_{2}(t){|}^{2}\leq \frac{1}{2}\ln(1+{ℳ}_{c}(\nu_{1},\nu_{2})).$$

*It implies that,*

$$b_{m}(\Theta \nu_{1},\Theta \nu_{2})\leq \frac{1}{2}\ln(1+{ℳ}_{c}(\nu_{1},\nu_{2})).$$ (14)

*Define* $\beta :{\mathbb{R}}_{+}\to \left[0,\frac{1}{2}\right)$ *by*

$$\beta (𝔓)=\frac{\ln(1+𝔓)}{2𝔓},\;\text{for}\;\;𝔓>0\;\;\text{and}\;\;\beta (0)\in \left[0,\frac{1}{2}\right).$$

*Thus, (* 14 *) becomes,*

$$b_{m}(\Theta \nu_{1},\Theta \nu_{2})\leq \left(\frac{\ln\left(1+{ℳ}_{c}((\nu_{1},\nu_{2})\right)}{2{ℳ}_{c}((\nu_{1},\nu_{2})}\right){ℳ}_{c}((\nu_{1},\nu_{2})\;=\beta ({ℳ}_{c}((\nu_{1},\nu_{2})){ℳ}_{c}((\nu_{1},\nu_{2}).$$

*Thus, all the necessities of Theorem 2.4 are true. So, the Eq (7) will have a Uni-Sol.* ◻

*Let us define* $d_{m}:{𝔐}_{b}\times {𝔐}_{b}\to {\mathbb{R}}_{+}$ *as follows*

$$d_{m}(𝔲,y)=|𝔲(t)-y(t)|,\;\text{for all }𝔲,y\in 𝔐.$$ (15)

*Evidently,* $\left(𝔐,d_{m}\right)$ *represent a complete metric space.*

**Theorem 3.4.** *Suppose the following presumptions are fulfilled:*

***(m)** The function* $\Gamma :[t_{0},\gamma ]\times [t_{0},\gamma ]\times \mathbb{R}\to \mathbb{R}$ *is continuous on* $\left[t_{0},\gamma \right]$, $[t_{0},\gamma {]}^{2}$, *and* $[t_{0},\gamma {]}^{2}\times \mathbb{R}$, *correspondingly.*
***(n)** For all* $\nu ,\alpha ,𝔣\in [t_{0},\gamma ]$ *and for all* 𝔲,y∈𝔐,
  $$|\Gamma (\nu_{1},\alpha_{1},{𝔣}_{1})\;-\Gamma_{1}(\nu_{2},\alpha_{2},{𝔣}_{2})\;|\leq \frac{M_{b}(\Theta \nu_{1},\Theta \nu_{2})-\varphi (M_{b}(\Theta \nu_{1},\Theta \nu_{2}))}{\left(1+t\right)},$$
*where* $M_{b}(\nu_{1},\nu_{2})$ and φ *are defined in Theorem 2.5.*

*Then, (7) will have a Uni-Sol.*

*Proof*: *Define a map* Θ:𝔐→𝔐

$$\Theta \nu (t)=\nu_{0}+\int_{0}^{t}\Gamma (\nu ,\alpha ,𝔣)\;d℘\;t\geq 0.$$ (16)

*The existence of a FP of* Θ *in (*16*) will ensure a Uni-Sol of the integral Eq (*7*).*

*Now, utilizing (15) and the above presumptions **(m)** and **(n)**,*

$$|\Theta \nu_{1}(t)-\Theta \nu_{2}(t)|=\left|\nu_{0}+\int_{0}^{t}\Gamma (\nu_{1},\alpha_{1},{𝔣}_{1})\;dt-\nu_{0}-\int_{0}^{t}\Gamma (\nu_{2},\alpha_{2},{𝔣}_{2})\;dt\right|\;=\left|\int_{0}^{t}(\Gamma (\nu_{1},\alpha_{1},{𝔣}_{1})-\Gamma (\nu_{2},\alpha_{2},{𝔣}_{2}))\;dt\right|\;\leq \frac{1}{\left(1+t\right)}\int_{0}^{t}(|\Gamma (\nu_{1},\alpha_{1},{𝔣}_{1})-\Gamma (\nu_{2},\alpha_{2},{𝔣}_{2})|)\;dt\;\leq \frac{M_{b}(\nu_{1},\nu_{2})-\varphi (M_{b}(\nu_{1},\nu_{2}))}{\left(1+t\right)}\int_{0}^{t}dt\;\leq M_{b}(\nu_{1},\nu_{2})-\varphi (M_{b}(\nu_{1},\nu_{2})).$$

*Further,*

$$\Theta \nu_{1}(t)-\Theta \nu_{2}(t)|\leq M_{b}(\nu_{1},\nu_{2})-\varphi (M_{b}(\nu_{1},\nu_{2})).$$

*Hence,*

$$d_{m}(\Theta \nu_{1},\Theta \nu_{2})\leq M_{b}(\nu_{1},\nu_{2})-\varphi (M_{b}(\nu_{1},\nu_{2})).$$

*Define* ψ:[0,∞)→[0,∞) *by*

ψ(𝔓)=𝔓.

$$\psi (d_{m}(\Theta \nu_{1},\Theta \nu_{2}))\leq \psi \left(M_{b}(\nu_{1},\nu_{2})-\varphi (M_{b}(\nu_{1},\nu_{2}))\right).$$

*Thus, all the necessities of Theorem 2.5 hold true. So, the Eq (7) will have a Uni-Sol.* ◻

**Example 3.1.** *Consider the climate model described by the integral equation*

$$\nu (t)=\nu_{0}+\int_{0}^{t}\left(\alpha \nu (\tau )\cos(\nu (\tau ))+\beta e^{-\tau }\right)d\tau ,\;t\in [0,T],$$

*where* ν(t) *represents the temperature anomaly,* α=0.3 *is the climate feedback parameter,* β=0.1 *models external forcing, and* $\nu_{0}=0$ *is the initial condition.*

*Let* $𝔐=C[t_{0},\gamma ]$ *represent set of all real-valued functions with no discontinuity on* [0,*T*], *endowed with the metric*

$$d_{m}(u,y)=\sup_{t\in [0,T]}|u(t)-y(t)|.$$

*Define the operator* Θ:𝔐→𝔐 *by*

$$(\Theta \nu )(t)=\int_{0}^{t}\left(0.3\nu (\tau )\cos(\nu (\tau ))+0.1e^{-\tau }\right)d\tau .$$

*To verify the conditions of Theorem 3.1, note that for all* u,y∈𝔐,

|Γ(u)−Γ(y)|=0.3|ucos(u)−ycos(y)|.

*Using the inequality* |ucos(u)−ycos(y)|≤2|u−y|, *it follows that*

|Γ(u)−Γ(y)|≤0.6|u−y|.

*Let* τ=−ln(0.6)>0 and define F(P)=ln(𝔚). *Then,*

$$d_{m}(\Theta u,\Theta y)\leq 0.6\;d_{m}(u,y)\;\Rightarrow \;F(d_{m}(\Theta u,\Theta y))\leq F(d_{m}(u,y))-\tau ,$$

*which satisfies the Ćirić-type contraction condition.*

*Moreover, the function*

$$\Gamma (\nu ,\alpha ,F)=0.3\nu \cos(\nu )+0.1e^{-t},$$

*is continuous in both* ν *and t*.

*Additionally, the growth condition*

$$|\Gamma (\nu_{1})-\Gamma (\nu_{2})|\leq 0.6|\nu_{1}-\nu_{2}|\leq \frac{M(u,y)}{e^{\tau }(1+t)},$$

*holds with M*(*u*,*y*) = *d*_*m*_(*u*,*y*), *since* $0.6<e^{-\tau }$ *for* τ=−ln(0.6).

*Therefore, all the conditions of Theorem 3.1 are true, and thus integral equation admits a unique solution* $\nu^{*}\in 𝔐$.

## 4 Existence results for Langevin equation

In this section, the same approach is utilized to investigate the stability of the Langevin equation.

$$\frac{dx}{dt}+\beta x=g(t),$$ (17)

where, *g*(*t*) is the external force, β is a positive constant (damping coefficient or stabilizing term).

Using the technique of green function, the Eq (17) can be transformed to

$$x(t)=\int_{0}^{t}K(t,s)g(s)ds+x_{0}(t),$$ (18)

where the Green function that describes how the system responds to the external forcing is

$$K=\left\{ \begin{array}{cc} e^{-\beta (t-s)}, & \text{if }t\geq s,\\ 0, & \text{if }t<s. \end{array}\right.$$

Let $𝔐=C[t_{0},\gamma ]$ represent set of all real-valued functions with no discontinuity. Let us define $d_{m}:𝔐\times 𝔐\to {\mathbb{R}}_{+}$ as

$$d_{m}(𝔲,y)=|𝔲(t)-y(t)|,\;\text{for all }𝔲,y\in 𝔐.$$ (19)

Evidently, $\left(𝔐,d_{m}\right)$ represents a complete 𝕄-space.

**Theorem 4.1.** *Assume the fulfillment of the following hypotheses:*

***(a2)** The function* g(t):[s,t]×[s,t]×ℝ→ℝ *is continuous on* $C[t_{0},\gamma ]$, $C[t_{0},\gamma {]}^{2}$, *and* $C[t_{0},\gamma {]}^{2}\times \mathbb{R}$, *correspondingly.*
***(a3)*** ∀ $s,t\in C[t_{0},\gamma ]$,
  $$|g(t)-g(s)|\leq e^{\tau }\frac{M(s,t)}{t-s}.$$
***(a4)*** ∀ $s,t\in C[t_{0},\gamma ]$,
  $$\int_{0}^{t}K(t,s)ds\leq \frac{t-s}{e^{2\tau }}.$$
*Where M*(*s*,*t*) *is defined in Definition 2.6 and* τ>0.

*Then the Eq (18) will have a unique solution.*

*Proof*: *Define a map* B:𝔐→𝔐

$$Bx(t)=\int_{0}^{t}K(t,s)g(s)ds+x_{0}(t).$$ (20)

*The existence of a FP of B in (* 20 *) will guarantee a Uni-Sol of the integral Eq (* 18 *).*

*Now utilizing (20) and the above presumptions **(a2)** to **(a3)***,

$$|Bx_{1}(t)-Bx_{2}(t)|=\left|x_{0}(t)+\int_{0}^{t}g(t)K(s,t)ds-x_{0}(t)-\int_{0}^{t}g(s)K(s,t)ds\right|\;=\left|\int_{0}^{t}K(t,s)(g(t)-g(s))ds\right|\;\leq e^{\tau }\frac{M(s,t)}{t-s}\int_{0}^{t}K(t,s)ds\;\leq \left|e^{\tau }\frac{\left(t-s\right)}{e^{2\tau }}\frac{M(s,t)}{\left(t-s\right)}\right|\;=e^{-\tau }M(s,t).$$

*This means that*

$$d_{m}(Bx_{1},Bx_{2})\leq e^{-\tau }M(s,t).$$

*Define* $ϝ:{\mathbb{R}}_{+}\to \mathbb{R}$ *by*

ϝ(𝔚)=ln(𝔚)for𝔚>0.

$$ϝ(d_{m}(Bx_{1},Bx_{2}))\leq ϝ(e^{-\tau }M(s,t))\;=\ln(M(s,t))-\tau \ln e.$$

*Implies that,*

$$\tau +ϝ(d_{m}(Bx_{1},Bx_{2}))\leq ϝ(M((s,t)))$$

*Thus, all the necessities of Theorem 2.1 hold true. So, the Eq (18) will have a Uni-Sol.* ◻

**Theorem 4.2.** *Consider that the subsequent assumptions are met:*

***(b2)** The function* g(t):[s,t]×[s,t]×ℝ→ℝ *is continuous on* $C[t_{0},\gamma ]$, $C[t_{0},\gamma {]}^{2}$, *and* $C[t_{0},\gamma {]}^{2}\times \mathbb{R}$, *correspondingly.*
***(b3)*** ∀ $s,t\in C[t_{0},\gamma ]$,
  $$|g(t)-g(s)|\leq e^{\tau }\frac{M_{a}(s,t)}{t-s}.$$
***(b4)*** ∀ $s,t\in C[t_{0},\gamma ]$,
  $$\int_{0}^{t}K(t,s)ds\leq \frac{t-s}{e^{2\tau }},$$
*where M*_*a*_(*s*,*t*) *is defined in Definition 2.7.*

*Then, (* 18 *) will have a Uni-Sol.*

*Proof*: *Define a map* B:𝔐→𝔐

$$Bx(t)=\int_{0}^{t}K(t,s)g(s)ds+x_{0}(t).$$ (21)

*The existence of a FP of B in (* 21 *) ensures a Uni-Sol of the integral Eq (* 18 *).*

*Now utilizing (21) and the above presumptions **(b2)** to **(b3)***,

$$|Bx_{1}(t)-Bx_{2}(t)|=\left|x_{0}(t)+\int_{0}^{t}g(t)K(s,t)ds-x_{0}(t)-\int_{0}^{t}g(s)K(s,t)ds\right|\;=\left|\int_{0}^{t}K(t,s)(g(t)-g(s))ds\right|\;\leq e^{\tau }\frac{M(s,t)}{t-s}\int_{0}^{t}K(t,s)ds\;\leq e^{\tau }\frac{\left(t-s\right)}{e^{2\tau }}\frac{M(s,t)}{\left(t-s\right)}\;=e^{-\tau }M(s,t).$$

*Hence,*

$$d_{m}(Bx_{1},Bx_{2})\leq e^{-\tau }M_{a}(s,t).$$

*Define* $ϝ:{\mathbb{R}}_{+}\to \mathbb{R}$ *by*

ϝ(𝔚)=ln(𝔚),for𝔚>0.

$$ϝ(d_{m}(Bx_{1},Bx_{2}))\leq ϝ(e^{-\tau }M_{a}(s,t))\;=\ln(M_{a}(s,t))-\tau \ln e.$$

*Therefore,*

$$\tau +ϝ(d_{m}(Bx_{1},Bx_{2}))\leq ϝ(M_{a}(s,t)).$$

*Next define* α:𝔐×𝔐→{−∞}∪[0,+∞) by

$$\alpha (s,t)=\left\{ \begin{array}{c} 1,\;\;if\;d_{m}(Bx_{1},Bx_{2})\geq 0,\\ -\infty ,\;else. \end{array}\right.$$

*It implies that,*

$$\tau +ϝ(d_{m}(Bx_{1},Bx_{2}))\leq \alpha (s,t)ϝ(M_{a}(s,t)).$$

*Thus, all the necessities of Theorem 2.2 hold true. Hence, the Eq (18) will have a Uni-Sol.* ◻

Let ${𝔐}_{b}=C[t_{0},\gamma ]$ represent set of all real-valued functions with no discontinuity and define $b_{m}:{𝔐}_{b}\times {𝔐}_{b}\to {\mathbb{R}}_{+}$ as

$$b_{m}(𝔲,y)=|𝔲(t)-y(t){|}^{2},\;\text{for all }𝔲,y\in {𝔐}_{b}.$$ (22)

Evidently, $\left({𝔐}_{b},b_{m},𝔟\right)$ is a complete *b*-metric space.

**Theorem 4.3.** *Assume the below presumptions fulfill:*

***(c1)** The function* g(t):[s,t]×[s,t]×ℝ→ℝ *defined to be continuous on* $C[t_{0},\gamma ]$, $C[t_{0},\gamma {]}^{2}$, *and* $C[t_{0},\gamma {]}^{2}\times \mathbb{R}$, *correspondingly.*
***(c2)** For all* $s,t\in C[t_{0},\gamma ]$
  $$|g(t)-g(s)|\leq \frac{\sqrt{\ln(1+{ℳ}_{c}(s,t))}}{\sqrt{𝔟}(t-s)}.$$
***(c3)*** ∀ $s,t\in C[t_{0},\gamma ]$,
  $$\int_{0}^{t}K(t,s)ds\leq (t-s),$$
*where* ${ℳ}_{c}(s,t)$ *is defined in Theorem 2.4.*

*Then, (18) will have a Uni-Sol and consequently will be stable.*

*Proof*: *Define a map* $B:{𝔐}_{b}\to {𝔐}_{b}$

$$Bx(t)=\int_{0}^{t}K(t,s)g(s)ds+x_{0}(t).$$ (23)

*The existence of a FP of B in (* 23 *) ensure a Uni-Sol of the integral Eq (* 18 *).*

*Now, utilizing (22) and the above presumptions **(c1)** to **(c3)***

$$|Bx_{1}(t)-Bx_{2}(t)|=\left|x_{0}(t)+\int_{0}^{t}g(t)K(s,t)ds-x_{0}(t)-\int_{0}^{t}g(s)K(s,t)ds\right|\;=\left|\int_{0}^{t}K(t,s)(g(t)-g(s))ds\right|\;\leq \frac{\sqrt{\ln(1+{ℳ}_{c}(s,t))}}{\sqrt{𝔟}(t-s)}\int_{0}^{t}K(t,s)ds\;=\frac{\sqrt{\ln(1+{ℳ}_{c}(s,t))}}{\sqrt{𝔟}(t-s)}(t-s).$$

*Further, since* 𝔟=2,

$$|Bx_{1}(t)-Bx_{2}(t){|}^{2}\leq \frac{1}{2}\ln(1+{ℳ}_{c}(s,t)).$$

*Accordingly,*

$$b_{m}(Bx_{1}(t)-Bx_{2}(t))\leq \frac{1}{2}\ln(1+{ℳ}_{c}(s,t)).$$ (24)

*Define* $\beta :{\mathbb{R}}_{+}\to \left[0,\frac{1}{2}\right)$ *by*

$$\beta (𝔓)=\frac{\ln(1+𝔓)}{2𝔓}\;\text{for}\;𝔓>0\;\text{and}\;\beta (0)\in \left[0,\frac{1}{2}\right).$$

*Thus, (* 24 *) becomes,*

$$b_{m}(Bx_{1}(t)-Bx_{2}(t))\leq \left(\frac{\ln\left(1+{ℳ}_{c}(s,t)\right)}{2{ℳ}_{c}(s,t)}\right){ℳ}_{c}(s,t).\;=\beta ({ℳ}_{c}(s,t)){ℳ}_{c}(s,t).$$

*Thus, all the necessities of Theorem 2.2 are true. So, the Eq (18) will have a Uni-Sol and consequently will be stable.*

Let $𝔐=C[t_{0},\gamma ]$ represent set of all real-valued functions with no discontinuity and define $d_{m}:𝔐\times 𝔐\to {\mathbb{R}}_{+}$ as

$$d_{m}(𝔲,y)=|𝔲(t)-y(t)|,\;\text{for all }𝔲,y\in 𝔐.$$ (25)

Evidently, $\left(𝔐,d_{m}\right)$ represent a complete metric space.

**Theorem 4.4.** *Assume the fulfillment of the following hypotheses:*

***(m1)** The function* g(t):[s,t]×[s,t]×ℝ→ℝ *is continuous on* $C[t_{0},\gamma ]$, $C[t_{0},\gamma {]}^{2}$, and $C[t_{0},\gamma {]}^{2}\times \mathbb{R}$, *correspondingly.*
***(m2)** For all* $s,t\in C[t_{0},\gamma ]$
  $$|g(t)-g(s)|\leq \frac{M_{b}(s,t)-\varphi (M_{b}(s,t))}{\left(t-s\right)}.$$
***(m3)*** ∀ $s,t\in C[t_{0},\gamma ]$,
  $$\int_{0}^{t}K(t,s)ds\leq (t-s).$$

*Then, (* 18 *) will have a Uni-Sol.*

*Proof*: *Define a map* B:𝔐→𝔐 *by*

$$Bx(t)=\int_{0}^{t}K(t,s)g(s)ds+x_{0}(t).$$ (26)

*The existence of a Uni-Sol of the integral Eq (* 18 *) is equivalent to the existence of a FP of B in (* 26 *).*

*Now, utilizing (26), (25) and the above presumptions **(m1)** - **(m2)***

$$|Bx_{1}(t)-Bx_{2}(t)|=\left|x_{0}(t)+\int_{0}^{t}g(t)K(s,t)ds-x_{0}(t)-\int_{0}^{t}g(s)K(s,t)ds\right|\;=\left|\int_{0}^{t}K(t,s)(g(t)-g(s))ds\right|\;\leq \frac{M_{b}(s,t)-\varphi (M_{b}(s,t))}{\left(t-s\right)}\int_{0}^{t}K(t,s)ds\;\leq \frac{M_{b}(s,t)-\varphi (M_{b}(s,t))}{\left(t-s\right)}(t-s).$$

*Further,*

$$|Bx_{1}(t)-Bx_{2}(t)|\leq M_{b}(s,t)-\varphi (M_{b}(s,t)).$$

*Hence,*

$$d_{m}(Bx_{1}(t),Bx_{2}(t))\leq M_{b}(s,t)-\varphi (M_{b}(s,t)).$$ (27)

*Define* ψ:[0,∞)→[0,∞) *by*

ψ(𝔓)=𝔓.

$$\psi (d_{m}(Bx_{1}(t),Bx_{2}(t)))\leq \psi (M_{b}(s,t)-\varphi (M_{b}(s,t)))$$

*Therefore, all the necessities of Theorem 2.5 hold true. So, the Eq (18) will have a Uni-Sol and consequently will be stable.* ◻

**Example 4.1.** *Let the integral equation:*

$$x(t)=\int_{0}^{t}K(t,s)g(s)\;ds+x_{0},$$

*where the Green’s function K*(*t*,*s*) *for* β=2 *is defined as*

$$K(t,s)=\left\{ \begin{array}{cc} e^{-2(t-s)}, & t\geq s,\\ 0, & t<s. \end{array}\right.$$

*Let* g(s)=sin(s), *and* $x_{0}\in \mathbb{R}$. *Then the equation becomes:*

$$x(t)=\int_{0}^{t}e^{-2(t-s)}\sin(s)\;ds+x_{0}.$$

*Define* 𝔐=C([0,T],ℝ), *the space of real-valued functions with no discontinuity on* [0,*T*], *endowed with the metric:*

$$d_{m}(u,v)=\sup_{t\in [0,T]}|u(t)-v(t)|.$$

*Define the operator* B:𝔐→𝔐 *by*

$$(Bx)(t)=\int_{0}^{t}e^{-2(t-s)}\sin(s)\;ds+x_{0}.$$

*For all* s,t∈[0,T],

$$|g(t)-g(s)|=|\sin(t)-\sin(s)|\leq |t-s|\leq \frac{M(s,t)}{e^{\tau }(1+t)},$$

*where M*(*s*,*t*) *is defined in Theorem 4.1 and we may take* τ=1.

$$\int_{0}^{t}e^{-2(t-s)}ds=\frac{1-e^{-2t}}{2}\leq \frac{t}{2}\leq \frac{t}{e^{-2}}=te^{2\tau },$$

*for* τ=1, *so the kernel satisfies the integral growth condition.*

*All conditions of Theorem 4.1 turn true. Therefore, the equation admits a unique solution* $x^{*}\in 𝔐$.

## 5 Conclusion

This paper investigates the stability of some climate systems models. In this direction, some important contractions such as *F*-contraction, α-*F*-contraction, rational type (ψ,ϕ)-contraction, and Geraghty type contraction have been utilized to formulate some novel existence results to ensure the stability of these models. The objective is to emphasize the fundamental characteristics that guarantee the practical effectiveness of these models in the disciplines of Meteorology and Climatology by conducting thorough analysis and applying fixed-point theorems from the existing literature. The uniqueness and existence of these models are significant for making sure that the models are accurate and reliable, which in turn is necessary for making smart decisions about climate change and disasters.
